# Treatment of Oral Multispecies Biofilms by an Anti-Biofilm Peptide

**DOI:** 10.1371/journal.pone.0132512

**Published:** 2015-07-13

**Authors:** Zhejun Wang, Cesar de la Fuente-Núñez, Ya Shen, Markus Haapasalo, Robert E. W. Hancock

**Affiliations:** 1 Division of Endodontics, Department of Oral Biological and Medical Sciences, Faculty of Dentistry, University of British Columbia, Vancouver, British Columbia, Canada; 2 Centre for Microbial Diseases and Immunity Research, Department of Microbiology and Immunology, University of British Columbia, Vancouver, Canada; 3 The State Key Laboratory Breeding Base of Basic Science of Stomatology (Hubei-MOST) & Key Laboratory of Oral Biomedicine Ministry of Education, School & Hospital of Stomatology, Wuhan University, 237 Luoyu Road, Wuhan, PR China; LSU Health Sciences Center School of Dentistry, UNITED STATES

## Abstract

Human oral biofilms are multispecies microbial communities that exhibit high resistance to antimicrobial agents. Dental plaque gives rise to highly prevalent and costly biofilm-related oral infections, which lead to caries or other types of oral infections. We investigated the ability of the recently identified anti-biofilm peptide 1018 to induce killing of bacterial cells present within oral multispecies biofilms. At 10 μg/ml (6.5 μM), peptide 1018 was able to significantly (p<0.05) prevent biofilm formation over 3 days. The activity of the peptide on preformed biofilms was found to be concentration-dependent since more than 60% of the total plaque biofilm cell population was killed by 10 μg/ml of peptide 1018 in 3 days, while at 5 μg/ml 50% of cells were dead and at 1 μg/ml the peptide triggered cell death in around 30% of the total bacterial population, as revealed by confocal microscopy. The presence of saliva did not affect peptide activity, since no statistically significant difference was found in the ability of peptide 1018 to kill oral biofilms using either saliva coated and non-saliva coated hydroxyapatite surfaces. Scanning electron microscopy experiments indicated that peptide 1018 induced cell lysis in plaque biofilms. Furthermore, combined treatment using peptide 1018 and chlorhexidine (CHX) increased the anti-biofilm activity of each compound compared to when these were used alone, resulting in >50% of the biofilm being killed and >35% being dispersed in only 3 minutes. Peptide 1018 may potentially be used by itself or in combination with CHX as a non-toxic and effective anti-biofilm agent for plaque disinfection in clinical dentistry.

## Introduction

Bacteria organized in multicellular biofilm communities pose a considerable clinical challenge as they cause more than 65% of all bacterial infections in humans, including oral diseases [1,2]. As one of the most complex biofilm systems in nature, human dental plaque causes a variety of oral infections including dental caries, pulp and periapical diseases [3,4]. Consequently, eradication of the microorganisms responsible for these infections is one of the primary goals in treatment [5,6]. Modern disinfecting agents have a limited number of macromolecular targets, such as essential bacterial proteins and membranes [7]. Due to the complex and heterogeneous organization of the microbial community [8], differential gene expression among cells within the biofilm, reduced growth/quiescence and the presence of extracellular polymeric substances, biofilms are quite recalcitrant to many antibiotics [9,10]. Therefore, there is an urgent need to develop novel anti-biofilm compounds and approaches that can overcome these challenges.

Anti-biofilm peptides have been recently identified as potential alternatives to traditional disinfecting agents due to their ability to specifically target bacterial biofilms, leading to the prevention of biofilm formation and dissolution of pre-existing biofilms in both Gram-negative and-positive bacterial pathogens [11–14]. In addition, peptides isolated from different microbial sources have shown anti-biofilm effects against different single-species oral biofilms, including *Streptococcus sanguinis* and *Enterococcus faecalis* biofilms [15,16]. Recent research has focused on optimizing cationic antimicrobial peptides that target planktonic bacteria [17,18]. However there is a major difference in structure-activity relationships for peptides that act against the biofilms [11,19]. Peptide 1018, originally isolated as an immunomodulatory peptide, was recently identified and characterized as a potent broad spectrum anti-biofilm compound that works by triggering the loss of the stress-signaling nucleotides, guanosine tetra- and penta- phosphates [(p)ppGpp], which appear to play an important role in biofilm development in multiple bacterial species [20]. It was demonstrated that the 1018 bound directly to ppGpp and acted in live bacterial cells to trigger degradation of this stress nucleotide [20]. Peptide 1018 has been shown to adopt different structures depending on its environment, and may be a promising candidate for the treatment of oral infections or as the active component in products (e.g. mouth rinse, composite resins, root canal sealers) used [21–23] for long-term dental treatment.

In the present study, we tested the effect(s) of anti-biofilm peptide 1018 against oral plaque biofilms grown in the presence and absence of saliva constituents to assess whether the peptide was suitable for use in dentistry settings. In addition, we evaluated the activity of the peptide in combination with the oral disinfectant chlorhexidine (CHX).

## Materials and Methods

### Peptide Synthesis

Peptide 1018 (VRLIVAVRIWRR) was synthesized by GenScript (Piscataway, NJ, USA) using solid-phase 9-fluorenylmethoxy carbonyl (Fmoc) chemistry and purified to a purity of >95% using reverse-phase high-performance liquid chromatography (HPLC) [20]. Peptide mass was confirmed by mass spectrometry. The peptide was resuspended in deionized water to make peptide stocks, from which peptide samples were taken and used in the present experiments; all stocks remained sterile over time.

### Minimal Inhibitory Concentration

The broth microdilution method with minor modifications for cationic peptides [24] was used for measuring the minimal inhibitory concentration (MIC) in brain-heart infusion (BHI) broth and lysogeny broth (LB) medium. The MIC was defined as the lowest concentration of peptide at which no growth was observed [11,24]. Planktonic cells from each of the three different dental clinical plaque samples were grown in different medium (BHI and LB). Peptide 1018 was dissolved in sterile water and stored in glass vials at 4°C. MIC assays were performed in sterile 96-well polypropylene microtiter plates. Peptide 1018 was added to the plates at increasing concentrations (0, 10, 20, 40, and 80 μg/ml), and plaque bacteria were inoculated to a final concentration of 5×10^5^ CFU/ml per well. The plates were incubated at 37°C for 24 hours. After 24 hours of peptide treatment, absorbance at 620 nm was measured using a microtiter plate reader (Bio-Tek Instruments Inc., VT, USA).

### Biofilm Model

Sterile hydroxyapatite (HA) disks (9.65 mm diameter by 1.52 mm thickness; Clarkson Chromatography Products, Williamsport, PA, USA) were used as the plaque biofilm substrate. Biofilms were formed on discs using a well-established model [25,26]. To allow for the formation of salivary pellicle, we also prepared saliva coated HA disk (sHA) by incubating each HA disk in a well of a sterile 24-well polystyrene cell culture plate (Corning, NY, USA) containing 400 μl infiltrated saliva for 4 hours. The saliva was collect from volunteers (at least 1.5 hours after meal) in sterile 14-mL polypropylene tubes (Corning, NY, USA) and filtrated by using sterilized 0.2 μm syringe filters.

The study was approved by the University of British Columbia Clinical Research ethics committee review boards (certificate H12-02430). Written informed consent has been obtained from the participants for collecting the saliva and plaque bacteria in this study. Supragingival plaque was collected from the first or second upper molars of each of three healthy adult volunteers (25–45 years old) and mixed in the same batch of BHI (Becton Dickinson, Sparks, MD, USA) by pipetting. The bacterial suspension was adjusted to optical density (OD) = 0.10, which was measured in 150 μl at 405 nm by a microplate reader (Model 3350; Bio-Rad Laboratories, Richmond, CA, USA) corresponding to 3.0×10^7^ CFU/ml as determined by serial tenfold dilutions and aerobic culturing on tryptic soy agar (TSA) plates for CFU counts. The HA and sHA disks were placed in the wells, each containing 1.8 ml of BHI, of a 24-well cell culture plate. Each well was inoculated with 0.2 ml of dispersed plaque suspension. The discs were incubated in the BHI-plaque suspension under anaerobic conditions (AnaeroGen; OXOID, Hampshire, UK) at 37°C for 3 days.

### Biofilm Inhibition Test

Three different concentrations of peptide (10 μg/ml, 5 μg/ml and 1 μg/ml) were added to the plaque suspension at the beginning of biofilm development, and maintained for 3 days (including 1, 2, and 3-day time intervals) under anaerobic incubation at 37°C. The control group consisted of adding sterile water into the culturing medium. Three HA and sHA disks were subjected to each group at each time interval.

### Long-term Anti-biofilm Effect of Peptide 1018 on Preformed Biofilms

Using the method mentioned in the Biofilm Model section above (without peptide), after the formation of a 3-day-old biofilm, the culture medium of each well was replaced by 1.98 ml of fresh BHI. Nine biofilm-covered HA and sHA disks were subjected to each of three different concentrations of peptide (10, 5, and 1 μg/ml corresponding to 6.5, 3.25 and 0.65 M respectively) in BHI. The first three HA and sHA disks from each peptide concentration were treated for 24 hours under anaerobic incubation at 37°C (24-hour treatment). Another three HA and sHA disks were treated a second time with the same concentration of peptide solution and cultured for another 24 hours (48-hour treatment), and the remaining three disks in each group were treated a third time and incubated for a third 24-hour period (72-hour treatment). A control condition with no peptide (only BHI + sterile water) was included for each time point evaluated (24, 48 and 72-hour).

### Short-term Anti-biofilm Effect of Peptide 1018 on Preformed Biofilms

Twelve 3-day-old plaque biofilm HA and sHA disks were prepared and rinsed in 2 ml of phosphate buffer saline (PBS) pH 7.0 (Sigma-Aldrich, St Louis, MO, USA) for 1 minute. Disks were then immersed in 1 ml of 10 μg/ml (6.5 μM) of peptide 1018 for 1 or 3 minutes for one or three times. Six disks treated by sterile water were set as the control group. Three disks were subjected to each group (e.g. 1 minute treatment for 3 times). Disks treated for three times were immersed in PBS for 1 minute between each treatment.

### Anti-biofilm Effect of Peptide 1018 in Combination with Chlorhexidine (Digluconate)

Twenty-four disks with 3-day-old plaque biofilm were prepared and rinsed in 2 ml of PBS for 1 minute. The disks were divided into four treatment groups: (i) Sterile water, (ii) 2% chlorhexidine digluconate (CHX), (iii) 10 μg/ml (6.5 μM) of peptide, and (iv) 2% CHX+ 10 μg/ml (6.5 μM) of peptide. Two percent CHX and 2% CHX+10 μg/ml peptide were freshly prepared from a 20% stock CHX solution (Sigma Chemical Co., St Louis, MO, USA) and a 5 mg/ml peptide 1018 stock solution, respectively. The HA and sHA disks were immersed in 2 ml solutions of each medicament for 1 or 3 minutes.

### Confocal Laser Scanning Microscopy Examination of Biofilm Samples Untreated or Treated with Peptide 1018 and/or Chlorhexidine

All plaque bacteria on HA disks that were exposed to the different treatments, as detailed above, were subjected to bacterial viability staining and confocal laser scanning microscopy. Disks were rinsed in 0.85% physiological saline for 1 minute before staining. LIVE/DEAD BacLight Bacterial Viability kit L-7012 for microscopy and quantitative assays (Molecular Probes, Eugene, OR, USA), containing two component dyes (SYTO 9 and propidium iodide in a 1:1 mixture) in solution, was used for staining the biofilm following the manufacturer’s instructions. The excitation/emission maxima for these dyes were 480/500 nm for the SYTO 9 whole cell stain and 490/635 nm for the dead cell stain propidium iodide. Fluorescence from each stained cell was viewed using a confocal laser scanning microscope (Nikon Eclipse C1; Nikon Canada, Mississauga, Ontario, Canada) at a 512 × 512 pixel scan area using a 20 × lens. Four random areas of the biofilm on each disk were scanned, resulting in 12 scanned areas for each group. A stack of 80–100 slices in 0.5 μm step sizes was captured from the top to the bottom of the biofilm. Confocal images were analyzed and quantitated (live/dead ratios) using the Imaris 7.2 software (Bitplane Inc., St Paul, MN, USA). The volume ratio of red fluorescence to green and red fluorescence indicated the proportion of killed cells. The actual killing effect was considered to be the difference between the ratio of dead bacterial volume following treatment and the same ratio in the sterile water control.

### Scanning Electron Microscopy Examination

Three additional plaque biofilms treated with 10 μg/ml (6.5 μM) of peptide 1018 for 24, 48 and 72 hours were collected for scanning electron microscopy (SEM) examination. Samples were prefixed with phosphate buffered 2.5% glutaraldehyde for 10 minutes before further fixation in 1% osmium tetroxide for 1 hour. The specimens were then subjected to increasing concentrations of ethanol (50%, 70%, 80%, and 100%) for dehydration. The dehydrated specimens were dried using a critical point drier (Samdri-795; Tousimis Research Corporation, Rockville, MD, USA), sputter-coated with gold palladium (Hummer VI; Technic Inc, Anaheim, CA, USA), and examined by SEM (Helios Nanolab 650, FEI, Eindhoven, Netherlands) at an accelerating voltage of 5 kV using 5,000 × and 20,000 × magnifications. A control experiment was done by preparing 10 μg/ml peptide in BHI solution and incubating it at 37°C for 72 hours. A droplet of the 72-hour 10 μg/ml of peptide was dropped on a piece of aluminum paper and air-dried for SEM imaging.

### Kill curves for Peptide 1018 treated *E*. *faecalis*, *S*. *mutans* and Plaque Biofilms

Bacterial strains *Enterococcus faecalis* VP3-181 and *Streptococcus mutans* NCTC 10449 were used. In addition, mixed plaque bacterial samples were collected from three healthy volunteers. The *E*. *faecalis* strain was grown overnight on blood agar plates (BHI agar with 5% heparinized sheep’s blood; Difco, Detroit, MI, USA) at 37°C in air. The *S*. *mutans* and plaque samples were grown overnight on blood agar plates at 37°C anaerobically. Bacterial suspensions were adjusted to optical density at 405 nm (OD_405_) of 0.10. *E*. *faecalis* and *S*. *mutans* were collected from the blood agar plates and mixed in both BHI and LB medium. To be consistent with the anti-biofilm assays using confocal microscopy, in this killing curves experiment, the plaque sample was collected from three healthy volunteers and biofilm was formed on the HA disk in the same way as described above in the Biofilm Model section.

The HA disks were placed in the 24-well plates, each containing 1.8 ml of BHI (or LB). Each well was inoculated with 0.2 ml of dispersed bacterial suspension (*E*. *faecalis*, *S*. *mutans* and plaque). The discs were incubated in the BHI (or LB)-bacteria suspension under anaerobic conditions (AnaeroGen; OXOID, Hampshire, UK) for *S*. *mutans* and plaque and under aerobic conditions for *E*. *faecalis*, at 37°C for 3 days. After 3 days, biofilms on HA disk surfaces were scraped off into BHI (or LB) medium using a plastic loop, followed by pipetting and vortexing. The suspension were adjusted to an OD_405_ of 0.25 for each bacterial species. A 100 μl sample of pure bacteria or the mixed plaque suspension were added to 400 μl of 10 μg/mL peptide 1018 solution in BHI (or LB) for 0, 30, 60 and 120 minutes. BHI (or LB) was used as control for each time intervals. At each of the indicated times of exposure, 100 μl of bacterial solution was added to 900 μl BHI (or LB) medium and diluted serially in 10-fold steps. Twenty μl from the dilution tubes was spotted onto blood agar plates. The blood agar plates with *S*. *mutans* and plaque were cultured anaerobically, and the *E*. *faecalis* plates were cultured aerobically, at 37 ˚C for 72 hours, and the colony forming unit (CFU) count was calculated. The number of CFU was generated as follows: (Number of bacterial colonies in one 20 μl droplet)×50×5×10^number of tenfold dilutions-1^. Three independent experiments were performed with three replicates each.

### Statistical Analysis

Statistical analysis was performed by SPSS 16.0 software (SPSS, Chicago, IL, USA) for Windows. Means and standard deviations of the proportions of dead cell volume and biofilm biovolume from confocal microscopy experiments were calculated respectively. Homogeneity of variance was determined using Levene’s test. Univariate ANOVA was applied and post hoc multiple comparisons were used to isolate and compare the significant results at a 5% significance level.

## Results

### Anti-biofilm Activity of Peptide 1018

**Fig 1:**
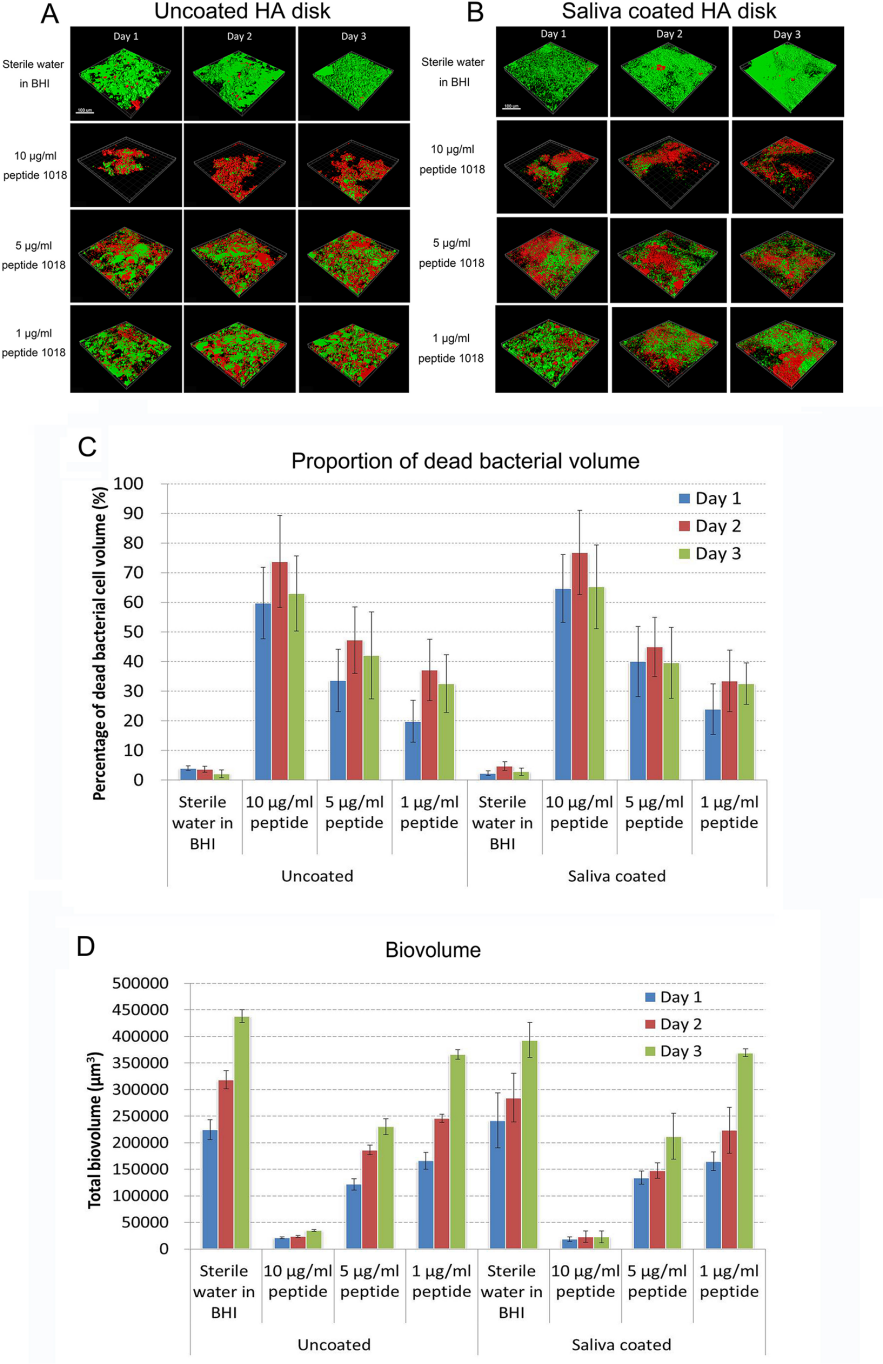
Peptide 1018 impacts on oral multispecies biofilm development. (A) Confocal microscopy images of plaque biofilm development in 3 days on uncoated HA disk surface with the addition of 10, 5 and 1 μg/ml peptide 1018. The scale bar represents 100 μm. (B) Confocal microscopy images of plaque biofilm development in 3 days on saliva coated HA disk surface with the addition of 10, 5 and 1 μg/ml peptide 1018. The scale bar represents 100 μm. (C) The proportion of dead biofilm bacterial cell volume during the 3-day biofilm development with the presence of peptide 1018. (D) Total biovolume of plaque biofilm formed in 3 days with the presence of peptide 1018.

Despite its modest antimicrobial activity against planktonic bacteria (based on MIC assays), peptide 1018 has been shown to be a potent inhibitor of biofilms produced by a wide range of bacterial species [22]. Following the established MIC method [24], we observed here that, at a concentration as high as 80 μg/ml (MIC>80 μg/ml), the peptide did not substantially inhibit planktonic growth of three independent dental plaque samples. This was further confirmed by measuring the absorbance (OD_620_) of planktonic plaque cultures treated with peptide 1018 in both BHI and LB medium (S1 Fig). In contrast, despite the usual 10–1000 fold resistance of biofilms to antibiotics [2], treatment with 10 μg/ml of peptide 1018 significantly and almost completely reduced plaque biofilm biovolume on hydroxyapatite (HA) and saliva-coated hydroxyapatite (sHA) disk surfaces by more than 10-fold after 72 hours of treatment, resulting in only 9%, 7% and 8% residual biofilm biovolume for HA groups, and 8%, 8% and 6% for sHA groups after 24, 48 and 72-hour time intervals, respectively, compared to the sterile water controls (p<0.05) (Fig 1A, 1B and 1D). Plaque biofilm with the addition of lower concentrations (5 and 1 μg/ml) of peptide formed 55±3% and 78±5% residual biovolume for HA groups (Fig 1A and 1D), 54±2% and 80±13% for sHA groups (Fig 1B and 1D), respectively. The total biovolume increased significantly (p<0.05) after 72 hours for all groups except for the 10 μg/ml peptide group (not significant). We postulated that the biofilm inhibitory effect of the peptide reflected the killing of bacteria within biofilms. Addition of 10 μg/ml of peptide 1018 at the beginning of biofilm development triggered the highest percentage of cell death (70% as assessed by propidium iodide uptake) after 48-hours of incubation among all groups (Fig 1A–1C). A similar increase in biofilm killing was observed in the 5 and 10 μg/ml groups between day 1 to day 2 followed by a slight decrease from day 2 to day 3 (Fig 1C). Significantly more microorganisms were killed in all experimental groups than in the water control at all time intervals tested (p<0.01).

**Fig 2:**
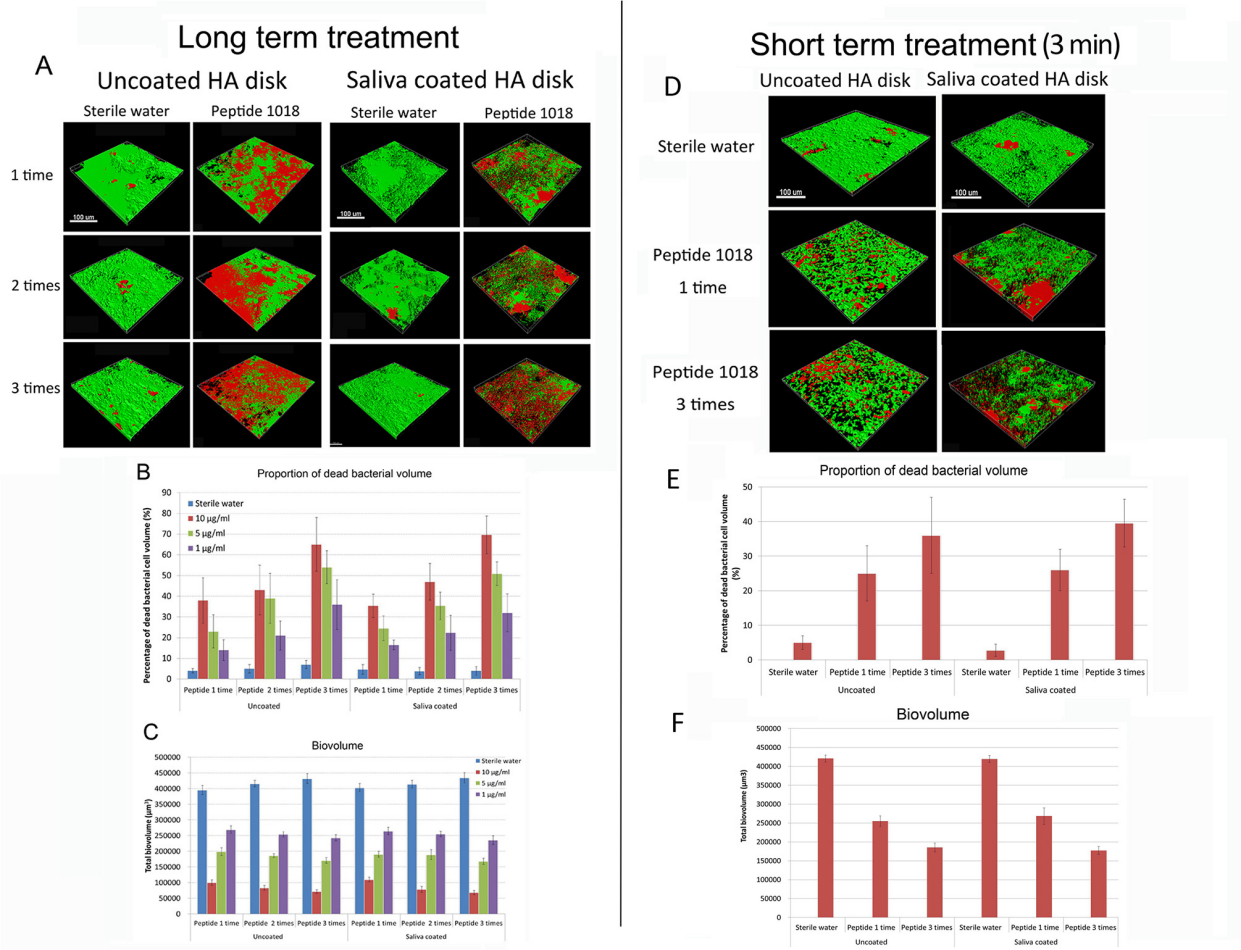
Peptide 1018 triggered cell lysis of 3-day-old oral multispecies biofilms. (A) Confocal microscopy images of 3-day-old plaque biofilms on HA and sHA surfaces treated with 10 μg/ml of peptide 1018. Samples treated 1 time were challenged with 1018 for 24 hours after biofilm formation for 3 days, biofilms treated twice were exposed to 1018 after day 3 for 2 more days, and peptide was added after day 3 for 3 additional days for samples treated 3 times. The scale bar represents 100 μm. (B) The proportion of dead biofilm bacterial cells after treatment with peptide 1018 once, twice or three times. (C) Total biovolume of biofilm after long-term peptide treatment. (D) Confocal microscopy images of 3-day-old plaque biofilms on HA and sHA surfaces treated once or three times with peptide 1018 for 3 minutes. The scale bar represents 100 μm. (E) The proportion of dead bacterial cells after different peptide treatments for 3 minutes. (F) Total biovolume of biofilm after short- term peptide treatment.

The peptide was also active against pre-formed biofilms (Fig 2). For example, in 3-day-old preformed biofilms, 3-time treatments with 10 μg/ml of peptide reduced biofilm biovolume to only 17% and 15% that of the untreated controls for HA and sHA surfaces respectively (Fig 2C). Additionally, one-time treatments suppressed biofilm formation by 4-fold (Fig 2C). Under all conditions tested, 5 μg/ml of peptide reduced the total biofilm biovolume to less than 50% of the untreated controls, while the lowest concentration of peptide tested (1 μg/ml) resulted in as low as 54% residual biovolume for sHA surface (Fig 2A and 2C). Moreover, peptide 1018 successfully killed preformed 3-day-old plaque biofilm bacteria both in long- and short-term treatments and both on HA and sHA surfaces (Fig 2). The number of killed biofilm bacteria correlated significantly with the time of exposure, the concentration of peptide used, and the frequency of medicament application (Fig 2B and 2E). All treatment groups showed a significant reduction of viable biofilm bacteria compared with the sterile water control group (p<0.05) (Fig 2A and 2B). Ten μg/ml of peptide 1018 applied to 3-day-old biofilms 3 times over 3 days killed the highest number of plaque biofilm bacteria (Fig 2B). The proportion of killed bacterial cells increased substantially with increasing concentrations of peptide 1018 in all cases (p<0.05) except when comparing groups treated twice with 10 and 5 μg/ml of peptide (not significant). The percentage of killed bacterial cells after three treatments (36–65% for biofilm on HA surface; 32–70% for biofilm on sHA surface) was significantly higher than after either one or two treatments (14–43% for biofilm on HA surface; 17–47% for biofilm on sHA surface) (p<0.05) (Fig 2A and 2B). Confocal microscopy examination showed no statistically significant difference in the 3-day bacterial killing, by peptide 1018, of pooled plaque from three donors compared to two separate plaque samples from two individual donors. For example, 10 μg/ml of peptide 1018 killed 66±15% (donor 1) and 64±17% (donor 2) plaque biofilm bacteria over 3 days (after treatment with peptide once every 24 hours for 72 hours), indicating no significant difference with the percentage value of biofilm killing of mixed plaque samples (Fig 2B).

**Fig 3:**
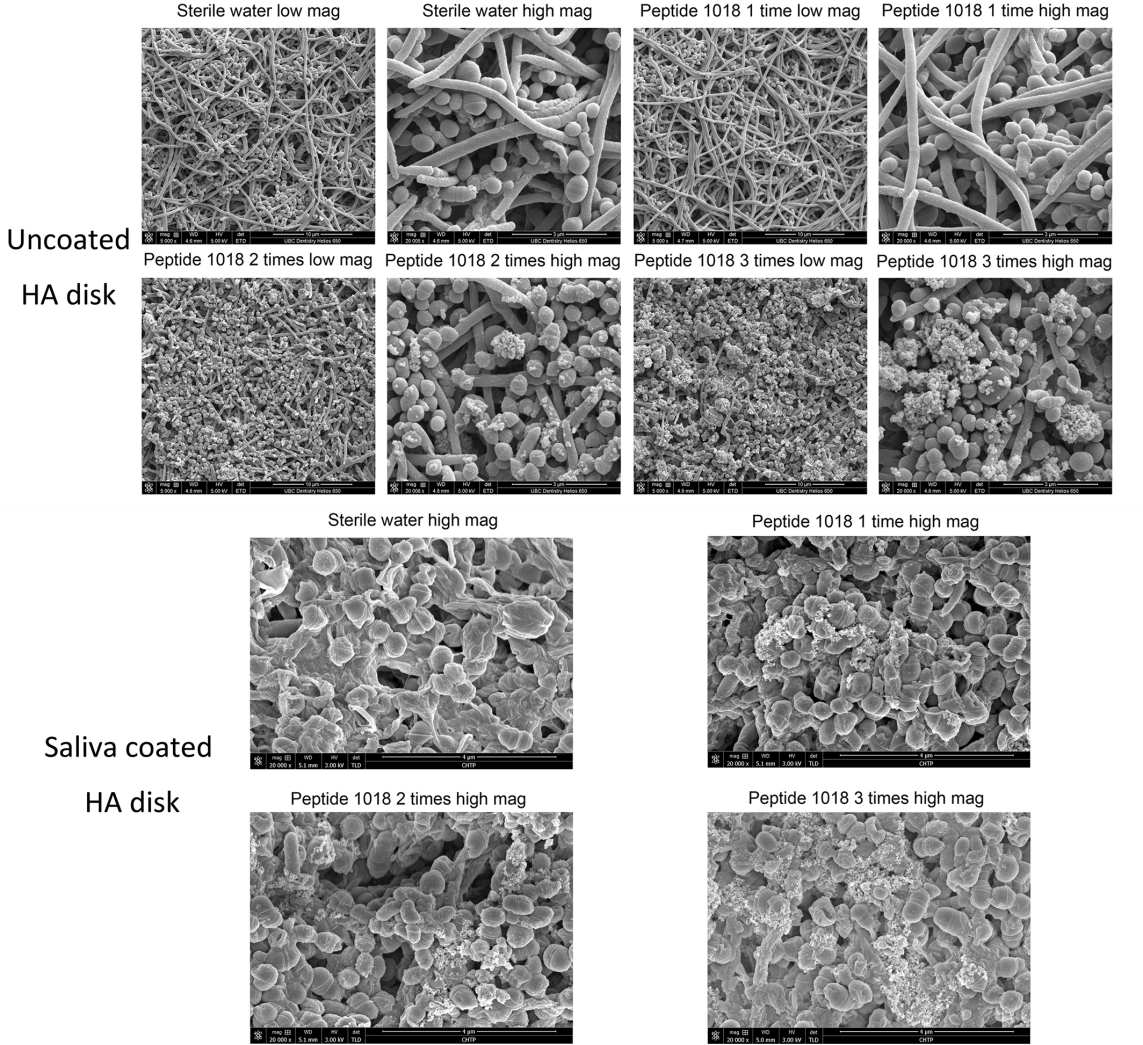
SEM micrographs showing cell killing of pre-formed plaque biofilms induced by different treatments with peptide 1018. The images show the effect of different treatments (one time, twice or three times) with 10 μg/ml of peptide 1018 on pre-formed (3-day old) plaque biofilms grown either on HA or saliva-coated HA disks. Peptide treated samples accumulated extracellular debris presumably from compromised cells; biofilm cells exhibited disrupted morphologies and were smaller in size in the treated samples. The low magnification corresponds to 5000 X, and the high magnification corresponds to 20 000 X.

We then performed short-term treatments to assess how rapidly peptide 1018 acted (Fig 2D, 2E and 2F). Under these experimental conditions, 3-minute treatments using 10 μg/ml of peptide reduced the biofilm biovolume to 44% (3 treatments) and 61% (1 treatment) on HA surfaces, 42% (3 treatments) and 64% (1 treatment) on sHA surfaces compared to water controls (Fig 3F). Moreover, 1-minute treatments (data not shown) using the same concentration of peptide decreased to 65% (3 treatments) and 68% (1 treatment) of the biofilm biovolume on HA surfaces, 59% (3 treatments) and 67% (1 treatment) on sHA surfaces. Each of these short term treatments led to statistically significantly lower biovolume values than the untreated controls (p<0.05). Again, reduced biovolume correlated with increased biofilm cell killing as confocal microscopy experiments revealed that short-term peptide treatments significantly triggered cell death in plaque biofilms by up to 39% (three 3-minute treatments of biofilms on a sHA surface) (Fig 2D and 2E), though the percentage of dead cells under these conditions was less than when biofilms were treated with peptide 1018 for longer periods of time.

The multispecies nature of the 3-day plaque biofilm was validated by SEM, showing cocci, rods and filaments within the biofilms that formed mixed communities (Fig 3). Killing of microorganisms present in plaque biofilms was further confirmed using this methodology. The control biofilms treated only with sterile water were well-organized network structures with smooth surfaces and virtually no dead (disrupted) bacterial cells. Cell lysis increased when biofilms were treated two and three times with peptide 1018, showing fine particles released within the biofilm structures (Fig 3). In addition, we show that the peptide does not aggregate under the conditions tested, as the control experiment with peptide 1018 only showed no aggregated peptide precipitation (S2 Fig).

**Fig 4:**
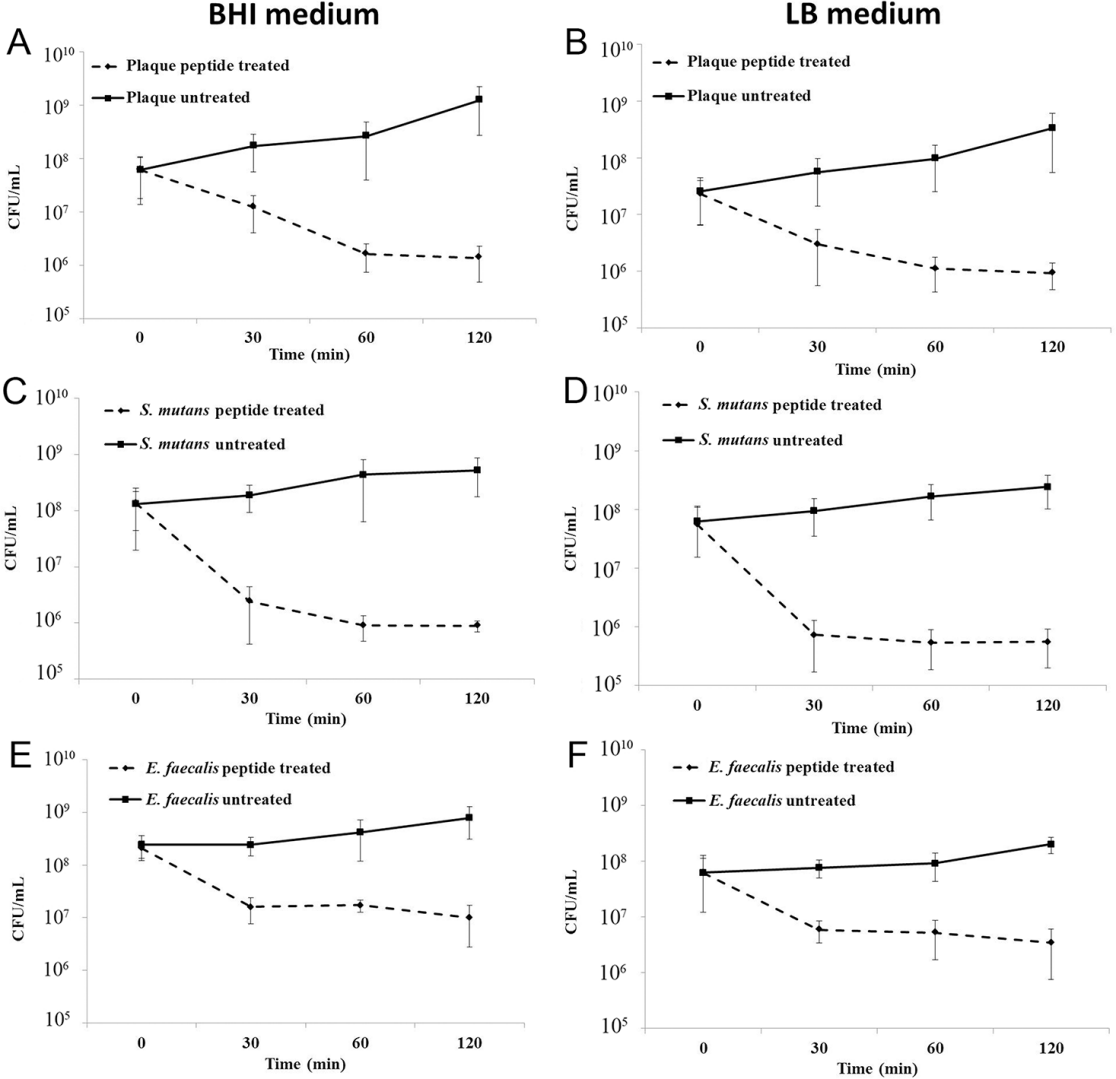
Peptide 1018 killed bacteria derived from dental plaque (A and B), *S*. *mutans* (C and D) and *E*. *faecalis* (E and F) biofilms grown on HA disks in BHI (A, C and E) and LB (B, D and F) medium. Cell killing was measured by performing CFU counts over time after dispersal of biofilms. Similar killing kinetics were observed using BHI and LB medium. The peptide killed bacteria harvested from biofilms 60 minutes post-treatment. No significant killing was observed after that. Error bars represent the standard deviations calculated from three independent experiments.

We further performed killing curves over a period of 120 minutes (Fig 4) to assess whether the peptide was indeed killing bacteria in plaque biofilms (Fig 4A and 4B). Bacteria were harvested from biofilms formed on HA disks cultured in BHI and LB medium and were treated with 10 μg/ml of peptide 1018. The peptide led to cell death in a time-dependent manner in both culture medium (Fig 4), with similar killing kinetics. We further tested whether peptide 1018 could kill bacteria derived from biofilms formed by individual bacterial species commonly found in the oral flora. Interestingly, the peptide led to substantial killing of *S*. *mutans* (Fig 4C and 4D) and *E*. *faecalis* (Fig 4E and 4F) biofilm cells, with *S*. *mutans* being more susceptible to the action of the peptide by killing over 90–99% bacteria at 30 to 120 minutes.

### Killing of Biofilms by the Combination of Anti-biofilm Peptide 1018 and CHX

**Fig 5:**
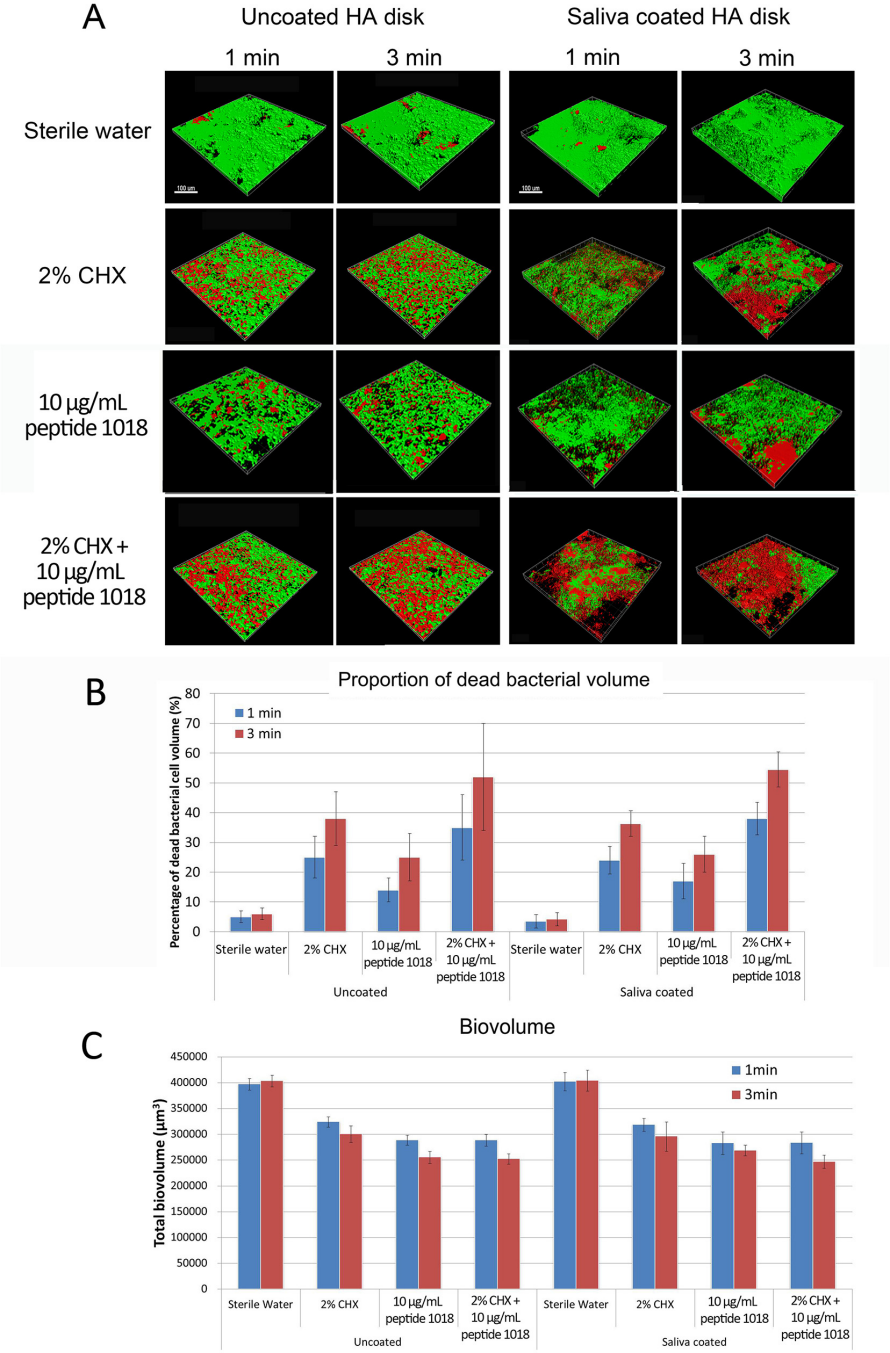
Combinations of peptide 1018 and CHX increased the anti-biofilm killing activity compared to each agent when used separately. (A) 3D Confocal microscopy images of 3-day-old plaque biofilms on HA and sHA surfaces treated with the combination of peptide 1018 and 2% CHX. The scale bar represents 100 μm. (B) The proportion of dead bacterial cells after treatment with peptide 1018, CHX or the combination of both agents. (C) Total biovolume of biofilm after treatments by peptide, CHX, and their combination.

Further, the short-term anti-biofilm effect of the peptide in combination with CHX was tested (Fig 5). The use of 10 μg/ml of peptide 1018 significantly reduced biofilm biovolume down to 62±4% (Fig 5C) while 2% CHX suppressed biovolume to 73±9% under 3-minute treatments, which were both significantly less than the sterile water control (p<0.05) (Fig 5C). However, although somewhat greater activity was observed there was no significant difference in residual biofilm biovolume was observed when the peptide was combined with CHX (Fig 5C). No statistically significant difference was found between HA and sHA surfaces for all of the above-tested biovolume comparisons.

Even though we had observed no difference in residual biofilm volume, the combined use of peptide 1018 and CHX showed a strong additive effect in bacterial killing (Fig 5A and 5B). In each case, the proportion of killed bacterial cells increased significantly with increasing time (1 and 3 minutes) of medicament exposure (CHX, peptide 1018 or CHX + peptide 1018) (Fig 5A and 5B). In addition, confocal microscopy consistently showed a higher percentage of dead cells in the combination groups compared to samples treated with either CHX or peptide 1018 alone (Fig 5A and 5B). Less than 6% of the entire biofilm cell population appeared dead in the sterile water control group (Fig 5B). No statistically significant difference was found between HA and sHA surfaces for any of the dead cell volume % comparisons described above.

## Discussion

Microorganisms from the oral environment are the primary etiologic agents of oral infections [15]. Dental plaque, which comprises diverse bacteria in the biofilm state, demonstrates enhanced resistance to antimicrobial agents [27]. Traditional disinfecting agents used against plaque biofilms are chemicals (e.g. CHX, sodium hypochlorite), which may inhibit biofilm development and affect bacterial metabolism [28]. Based on our previous investigations [25,26], the biofilm model described in the present study provides a method for the *in vitro* study of multispecies biofilms that closely mimic *in vivo* biofilms. Here we included an extra saliva-coated HA disks model for the purpose of mimicking the interaction between the diverse microbial community in the oral cavity and the proteinaceous film, known as the saliva pellicle, present on the tooth surface.

Recently developed and identified synthetic anti-biofilm peptides offer an alternative approach to combat biofilms [11,13,16]. The present study demonstrated the potent anti-biofilm activity of a short synthetic amphiphilic peptide, 1018, on oral plaque biofilms in terms of inhibiting biofilm development and stimulating killing of organisms in the biofilm. This broad spectrum anti-biofilm peptide has previously been shown to act by binding to and stimulating degradation the second messenger nucleotide (p)ppGpp that is involved in biofilm formation and maintenance [20]. Here, we showed that 1018 triggered cell death of 3-day old plaque biofilms at concentrations well below the MIC (>80 μg/ml) (S1 Fig). Indeed 10 μg/ml of peptide 1018 successfully inhibited plaque biofilm formation by suppressing more than 75% of biofilm growth quantified as biofilm biovolume compared to the water control group (Fig 1). This is consistent with broad spectrum killing and dispersal activity vs. plaque biofilm bacteria (Figs 1 and 2), consistent with the previously demonstrated activity vs. diverse Gram negative and positive bacteria [20], since plaque biofilms are known to contain both Gram-negative and Gram-positive bacterial cells [28]. However, the specific bacterial species present in the different inocula from dental plaque samples and the residual bacteria after treatment were not identified. Further studies will focus on isolating these species and exploring the metabolic diversity of oral multispecies biofilms. As no significant difference was found in either biovolume or percentage of dead cell volume in the biofilm inhibition and killing tests in the present study between HA and sHA surfaces, we conclude that the saliva pellicle most likely does not degrade peptide 1018. These results are substantially different from those obtained with another antimicrobial peptide LL-37, for which antimicrobial activity was reduced by saliva and other body fluids [29]. Also of importance to its potential applicability, peptide 1018 has been demonstrated to be nontoxic at 200 μg/ml vs. human fibroblasts and at 375 μg/ml vs. human red blood cells [22,30].

Chlorhexidine is a cationic bisbiguanide with broad antibacterial activity and has become one of the most frequently-used disinfectants in oral antimicrobial strategies [31]. Chlorhexidine reacts with negatively charged groups on the cell envelope [32], causing an irreversible loss of cytoplasmic constituents, membrane damage, and enzyme inhibition [26]. Previous studies have shown that 2% CHX (20 μg/ml) was able to kill 46±3% of plaque biofilm formed on a hydroxyapatite disk within 3 minutes [25,33], an activity that is less than that showed in these studies by peptide 1018. Moreover, the antimicrobial action of CHX alone is nonspecific, and its use at concentrations that are effective at eliminating biofilms is associated with side effects such as staining of teeth and changes in taste perception [34]. Kim *et al*. [12] reported a synergistic inhibitory effect of cationic peptides and CHX on the planktonic growth of oral streptococci using MIC test. Therefore we asked whether peptide 1018, aside from its potent anti-biofilm properties, was compatible with and/or could act in combination with CHX to treat established plaque biofilms. Interestingly, while the combination of peptide 1018 and CHX did not significantly reduce biofilm biovolume in comparison with the activity of each agent when used alone (Fig 5C), the combined treatment led to a significant increase in the amount of dead cells within biofilms (Fig 5B). In addition, the saliva coating did not interfere with the antimicrobial activity of CHX and its combination with peptide 1018.

There are some experimental limitations associated with the method utilized here. For instance, it is likely that not all species present in plaque samples isolated from volunteers will grow well *in vitro*, as some of them may be difficult to culture. Peptide 1018 may not be active against all microorganisms present in the oral flora, and will likely be more potent against specific species, as for example seen here for *S*. *mutans* compared to *E*. *faecalis* (Fig 4). It is also important to note that performing killing assays by CFU counting using biofilm bacteria is limited by the fact that such assays involve handling bacteria in suspension, which are no longer surface-associated and therefore likely not biofilm bacteria *per se*. Indeed, we observed peptide-induced bacterial killing for the first 60 minutes of the experiment but not afterwards. In addition, we do not as yet know whether the biofilm inhibitory effect of the peptide is preserved in long-term experiments (several weeks), or whether the peptide would be active against older biofilms that are associated e.g. with endodontic and periodontal infections. Based on our experiments, we anticipate that the peptide would have to be applied multiple times to achieve optimal results. Nevertheless, the peptide holds promise since, in clinical dental settings, it could potentially be used as an active ingredient of toothpaste, mouthwash or chewing gum to prevent oral infections. Indeed, the peptide showed good anti-biofilm activity within 3 minutes and is therefore suitable for such applications.

Live/Dead viability staining was used for the CLSM experiments. Viability staining is based on the principle that the red stain (propidium iodide) enters only those cells where the cell membrane is damaged, whereas the green stain (SYTO 9) can enter all cells. It is therefore possible that in some cases red fluorescence may give a false-positive result when interpreted as a killed cell, in the case that the cell is still alive although damaged. Despite its shortcomings, viability staining has become the method of choice in measuring biofilm killing [33]. The methodology allows for the measurement of the relative proportion of killed bacteria in each biofilm specimen, which was not possible by using traditional culture methods.

In conclusion, with its potent, stand-alone anti-biofilm activity, and its biofilm killing effects in combination with CHX, peptide 1018 appears to be a promising candidate for antimicrobial therapy against oral biofilm infections. While mechanical removal is still necessary to control daily plaque biofilm growth, the application of peptide 1018 used alone or in combination with CHX may contribute to the efficient control of oral biofilm growth *in vivo*.

## Supporting Information

S1 Fig Effect of increasing concentrations of peptide 1018 on planktonic growth of plaque samples grown in BHI and LB medium after 24 hours. Bacteria from plaque samples were grown in BHI and LB medium using 96-well polypropylene plates in the presence of increasing concentrations of peptide 1018 and planktonic growth (measured absorbance at 620 nm) was assessed after 24 hours.
(DOCX)

S2 Fig SEM micrograph showing the absence of aggregation of peptide 1018 (10 μg/ml) in BHI solution. 10 μg/ml peptide solution was prepared in BHI solution and incubated at 37°C for 72 hours. A droplet of the 72-hour 10 μg/ml of peptide was dropped on a piece of aluminum paper and air-dried.
(DOCX)
