# Supplementary material for: Treatment of Oral Multispecies Biofilms by an Anti-Biofilm Peptide
Source: PLoS One. 2015 Jul 13;10(7):e0132512. doi: 10.1371/journal.pone.0132512 (PMC4500547; doi:10.1371/journal.pone.0132512)
Supplement: S1 Fig — Bacteria from plaque samples were grown in BHI and LB medium using 96-well polypropylene plates in the presence of increasing concentrations of peptide 1018 and planktonic growth (measured absorbance at 620 nm) was assessed after 24 hours. (DOCX) [file pone.0132512.s001.docx]

**Supporting Information 1**

**
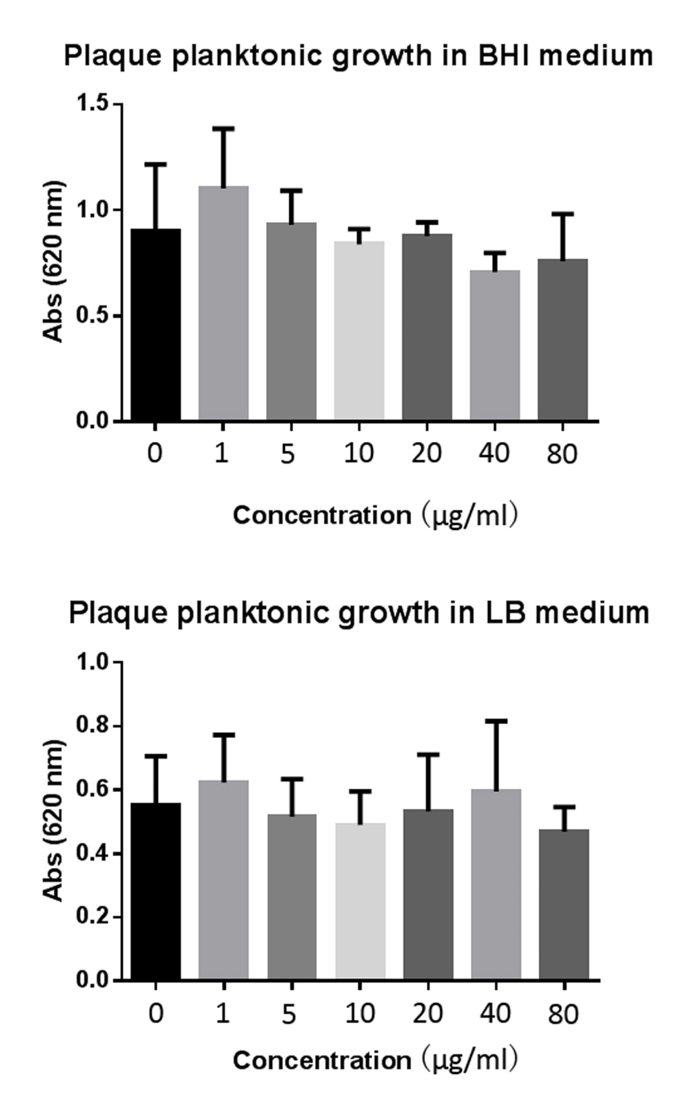
**

**S1 Fig.** **Effect of increasing concentrations of peptide 1018 on planktonic growth of plaque samples grown in BHI and LB medium after 24 hours.** Bacteria from plaque samples were grown in BHI and LB medium using 96-well polypropylene plates in the presence of increasing concentrations of peptide 1018 and planktonic growth (measured absorbance at 620 nm) was assessed after 24 hours.
