# Supplementary material for: Treatment of Oral Multispecies Biofilms by an Anti-Biofilm Peptide
Source: PLoS One. 2015 Jul 13;10(7):e0132512. doi: 10.1371/journal.pone.0132512 (PMC4500547; doi:10.1371/journal.pone.0132512)
Supplement: S2 Fig — 10 μg/ml peptide solution was prepared in BHI solution and incubated at 37°C for 72 hours. A droplet of the 72-hour 10 μg/ml of peptide was dropped on a piece of aluminum paper and air-dried. (DOCX) [file pone.0132512.s002.docx]

**Supporting Information 2**

**
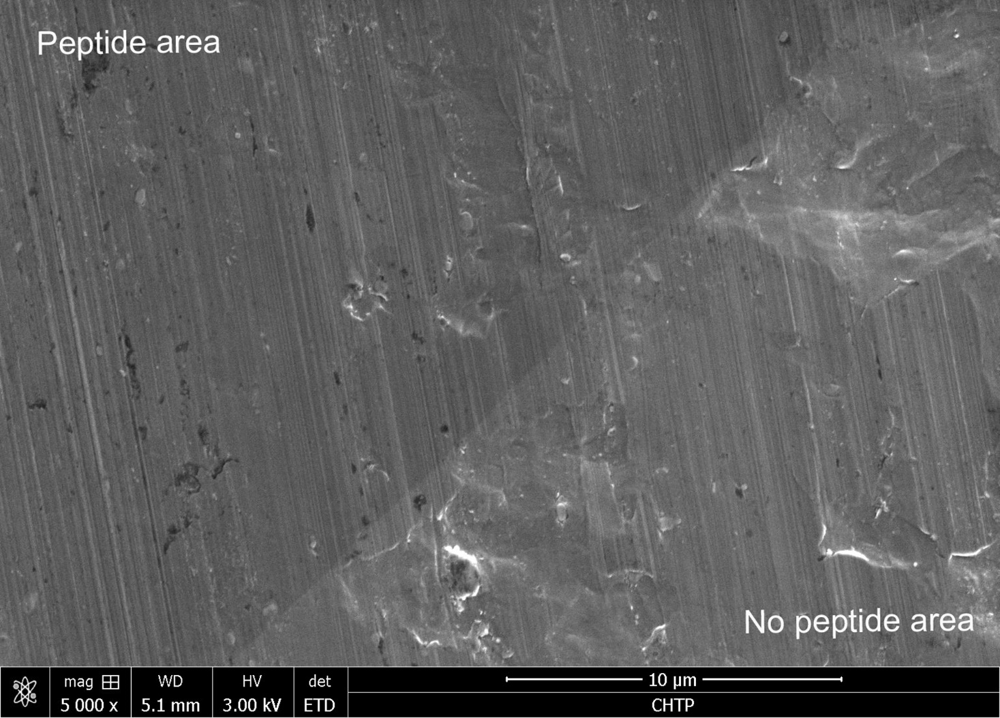
**

**S2 Fig. SEM micrograph showing the absence of aggregation of peptide 1018 (10 μg/ml) in BHI solution.** 10 μg/ml peptide solution was prepared in BHI solution and incubated at 37˚C for 72 hours. A droplet of the 72-hour 10 μg/ml of peptide was dropped on a piece of aluminum paper and air-dried.
